# Microneedle-guided monitoring and therapy in chronic limb-threatening ischemia

**DOI:** 10.3389/fcell.2026.1824311

**Published:** 2026-04-30

**Authors:** Zhen Zhang, Xiao-Dan Zhao, Aikeremujiang Ahamujiang, Yan-Hong Wang, Cheng Long, Guanglin Wang

**Affiliations:** 1 Trauma Medical Center, Department of Orthopedic Surgery, West China Hospital, Sichuan University, Chengdu, China; 2 Department of Orthopedic, Yili Prefecture Friendship Hospital, Yining, China; 3 Department of Rehabilitation Medicine, West China Second University Hospital of Sichuan University (WCSUH-SCU), Sichuan University, Chengdu, China

**Keywords:** chronic limb-threatening ischemia, drug delivery, interstitial fluid biosensing, microneedle, wound monitoring

## Abstract

Chronic limb-threatening ischemia (CLTI) represents the most severe form of peripheral artery disease, with high risks of infection, tissue loss, amputation, and mortality. Continuous or high-frequency monitoring could improve early detection of deterioration between clinic visits, yet current perfusion tests are limited by variability and episodic use. This mini review focuses on microneedle-enabled access to interstitial fluid for trend-based monitoring and local therapy in CLTI. Evidence is synthesized linking oxygenation, lactate, and pH to ischemic pathophysiology and infection risk, and microneedle sensing approaches for these core markers are outlined. Local delivery design patterns that support staged care—stabilizing the microenvironment, controlling infection, and promoting regeneration—are summarized while maintaining sensing interpretability. Key translation barriers include drift and calibration during chronic wear, safety in fragile ischemic skin, and integration with clinical workflows. Minimally sufficient, trend-oriented sensing combined with phase-matched local interventions provides a pragmatic pathway toward clinically actionable “sense–treat” microneedle systems for CLTI.

## 1 Introduction

Peripheral artery disease (PAD) affects hundreds of millions of people worldwide, and its burden has continued to increase over recent decades (GBD, 2019 Peripheral Artery Disease Collaborators, 2023). Chronic limb-threatening ischemia (CLTI) is the most severe manifestation of PAD. It typically presents with ischemic rest pain, nonhealing ulceration, or gangrene, and it confers a high risk of major amputation and death (Conte et al., 2019). Despite advances in contemporary care, CLTI remains a high-cost, high-morbidity syndrome. This burden is greatest when tissue loss co-occurs with infection and wound management becomes prolonged and resource demanding (Duff et al., 2019). In chronic ischemic wounds, microbial biofilms are increasingly recognized as major barriers to healing. They sustain inflammation, impair granulation and epithelialization, and reduce susceptibility to antimicrobials and host defenses (Metc et al., 2013).

In clinical practice, first-line hemodynamic screening, such as the ankle–brachial index, can be misleading in patients with diabetes or chronic kidney disease. Medial arterial calcification and vessel noncompressibility may confound interpretation (Abu et al., 2020). Multiple perfusion and oxygenation tests have been evaluated for outcome prediction in diabetic foot and ischemic ulcer care, including transcutaneous oxygen pressure, toe pressures, skin perfusion pressure, and hyperspectral measures. However, the comparative quality of evidence and the consistency of thresholds remain limited (Wang et al., 2016). Ischemic deterioration often progresses between clinic visits. This creates substantial interest in continuous or high-frequency monitoring approaches that can identify early physiological changes before clinically apparent tissue breakdown (Houghton et al., 2013).

Interstitial fluid (ISF) is a biofluid that provides clinically useful information and has a composition related to blood, and wearable ISF biosensors are emerging as a minimally invasive route for longitudinal, decentralized monitoring (Wu et al., 2024). Microneedles are tiny, skin-friendly needles that are typically fabricated as a small patch or array. They gently penetrate only the outer layers of skin to interface with the tissue just beneath the surface, usually with minimal discomfort. Microneedle platforms are well suited to this application because they can access dermal ISF while remaining shallow, modular, and compatible with on-skin form factors that enable integrated sensing and therapy (Barnum et al., 2020). Proof-of-concept microneedle electrochemical sensing has been demonstrated for clinically relevant microenvironmental signals such as pH, including use to characterize ischemia-associated changes in a peripheral artery disease model (Lee et al., 2021). Similarly, microneedle sensor patches have enabled intradermal and ISF lactate monitoring with wearable readouts, supporting the feasibility of tracking ischemia–metabolism coupling over time (Wang et al., 2024). Optical microneedle arrays have also been developed to measure tissue oxygen partial pressure, highlighting a path toward direct oxygenation sensing beyond surface-limited methods (Müller et al., 2022).

This mini review examines microneedle-enabled monitoring and local biomaterial-mediated treatment for limb ischemia. It explicitly addresses infection risk and regenerative outcomes, including soft tissue repair, revascularization, and barrier restoration. Accordingly, this review prioritizes pH, lactate, and tissue oxygenation as core sensing targets. The following sections relate these markers to CLTI pathophysiology and summarize microneedle-based monitoring and local therapy design patterns that may support infection control and regeneration.

## 2 Pathophysiology of CLTI relevant to microneedle design

### 2.1 Hypoperfusion and hypoxia reshape repair capacity

**FIGURE 1:**
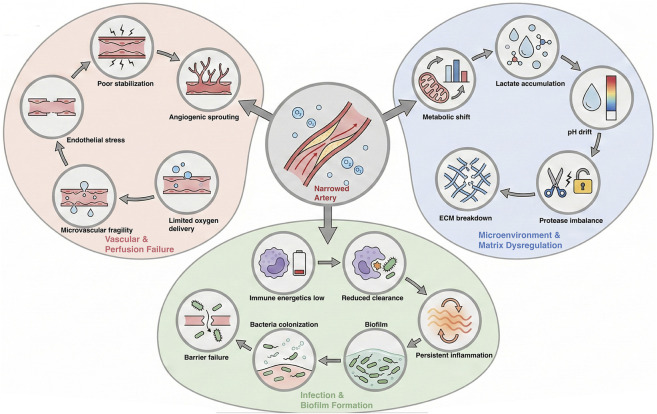
CLTI pathophysiology as a clustered, state-driven framework. The schematic organizes chronic limb-threatening ischemia into three interacting domains—vascular/perfusion failure, microenvironment and matrix dysregulation, and infection/biofilm with immune impairment—each represented as an internal causal cascade. A central hypoperfusion–hypoxia hub provides the common upstream constraint and links to each domain, emphasizing that persistent oxygen delivery deficit shapes downstream vascular instability, metabolic/chemical drift with protease-mediated extracellular matrix breakdown, and impaired host defense that facilitates colonization and biofilm persistence. Within-cluster arrows depict the dominant progression of processes in each domain, highlighting how CLTI behaves as a multi-state chronic system rather than a single-threshold phenomenon.

CLTI is fundamentally a state of sustained hypoperfusion. In this state, oxygen delivery does not meet tissue metabolic demand, which establishes a chronic hypoxic niche that differs qualitatively from the transient hypoxia of acute healing (Figure 1) (Guo and DiPietro, 2010). Chronic hypoxia can impair keratinocyte re-epithelialization. Migration programs may be disrupted, and the wound edge may remain “stalled” despite adequate debridement and topical care (Xia et al., 2001). Ischemic hypoxia can also induce regulatory microRNAs such as miR-210 that directly attenuate epithelial closure in ischemic wound models, providing a mechanistic link between low oxygen tension and delayed re-epithelialization (Biswas et al., 2010). Beyond the epidermis, hypoxia can compromise fibroblast-to-myofibroblast transition and contractile function. This weakens mechanical closure and matrix remodeling that are required for stable wound contraction (Modarressi et al., 2010).

At the vascular level, hypoxic signaling is a double-edged regulator of repair. It can initiate angiogenic programs, yet it may still fail to restore a mature microvascular network when perfusion cannot be re-established (Nauta et al., 2014). Inadequate vascular stabilization, including impaired pericyte–endothelial coordination, can limit durable revascularization and prolong tissue vulnerability even when early sprouting is observed (Bodnar et al., 2016). Oxygen delivery also constrains immune cell energetics and effector function. As a result, hypoperfusion can weaken local microbial clearance and increase susceptibility to infection during tissue loss (Hong et al., 2014). Clinically, oxygen-related measurements such as transcutaneous oxygen pressure ( ${\text{TcPO}}_{2}$ ) are widely used to estimate tissue viability and healing potential. This use reinforces oxygenation as a practical prognostic variable in limb salvage pathways (Leenstra et al., 2020). However, ${\text{TcPO}}_{2}$ values and thresholds vary across studies and are influenced by measurement conditions. This variation supports interest in repeated and trend-based oxygenation readouts rather than isolated single-timepoint interpretation (Catella et al., 2021).

### 2.2 Metabolic stress and microenvironment drift

Hypoxia promotes a shift toward glycolysis with lactate accumulation in wounded tissues, and lactate has been shown to stimulate angiogenesis and accelerate wound closure in superficial and ischemic settings (Porporato et al., 2012). Earlier work further suggested that lactate is not merely a waste product but a signaling intermediate that can promote collagen deposition and revascularization even under aerobic conditions (Hunt et al., 2007). Chronic wounds also commonly exhibit altered pH trajectories that often drift toward alkalinity, and this pattern correlates with slower healing and poorer prognosis in clinical wound environments (?). Wound pH is mechanistically relevant because it modulates matrix metalloproteinase (MMP) activity and inhibitor balance. In doing so, it shapes extracellular matrix turnover and the capacity of a wound to transition into productive remodeling (Percival et al., 2014). Consistent with this framework, chronic wounds often show persistently elevated MMP-9 levels, and experimental evidence supports a causal contribution of MMP-9 to delayed healing phenotypes (Reiss et al., 2010). The pH milieu also influences antimicrobial performance and microbial ecology, which is clinically relevant when ischemic wounds require prolonged topical or systemic antibiotic exposure (Jones et al., 2015). From a systems perspective, these metabolic and chemical drifts in lactate and pH are best interpreted as “state variables” that couple perfusion deficit to immune dysfunction, protease imbalance, and biofilm permissiveness (Holzer-Geissler et al., 2022).

### 2.3 Infection and biofilm as state accelerators

Ischemic tissue loss creates a permissive substrate for microbial colonization, and biofilm formation is increasingly recognized as a dominant barrier to healing in chronic wounds. In experimental diabetic wound models, biofilm-challenged wounds show delayed closure, deficient vascularization, and sustained inflammatory cytokine expression. These findings indicate that infection can reshape the overall healing trajectory rather than simply add “bioburden” (Zhao et al., 2012). In addition, *Staphylococcus aureus* biofilm infection can compromise wound healing by reducing granulation tissue collagen. This links biofilm state to impaired tissue biomechanics and increased vulnerability to recurrence (Roy et al., 2020). Clinical data in CLTI populations further support that the presence and severity of foot infection are independently associated with amputation risk. This emphasizes infection as a key modifier of outcomes rather than a secondary complication (Salm et al., 2024). In diabetic foot infection cohorts, major complications frequently include surgical amputation and prolonged antimicrobial exposure. This highlights the need to integrate infection control into regenerative strategies for ischemic limbs (Uysal et al., 2017).

### 2.4 Regeneration is constrained by timing and state transitions

Cutaneous wound repair proceeds through coordinated phases, and failure to transition from inflammation to proliferative rebuilding is a hallmark of chronic non-healing states (?). Inflammation must be actively resolved after danger signals are cleared. Otherwise, persistent activation of innate immune programs can lock wounds into a destructive loop that prevents regeneration (Landén et al., 2016). A molecular view indicates that inflammatory mediators are not uniformly detrimental. Instead, their magnitude and duration shape whether repair becomes regenerative or fibrotic and pathologic (Eming et al., 2007). Accordingly, therapeutic strategies for ischemic ulcers should be staged. They should first stabilize oxygen debt and microenvironmental stress, then control infection and collateral inflammatory injury, and only then amplify pro-regenerative programs that require a permissive niche (Han et al., 2022).

Collectively, these coupled state variables—oxygenation, lactate and pH drift, protease imbalance, and biofilm-accelerated inflammation—support the selection of microneedle-accessible biomarkers, including pH, lactate, and tissue oxygenation (Wu et al., 2024). They also inform the timing and composition of local biomaterial-delivered interventions (Wu et al., 2024).

## 3 Microneedle-enabled monitoring

**FIGURE 2:**
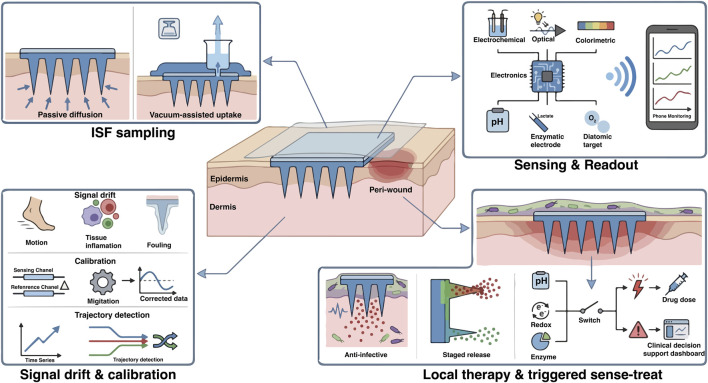
Microneedle (MN) patch functions for CLTI wound care: sampling, monitoring, drift mitigation, and triggered local therapy. A MN patch interfaces with dermal tissue to enable minimally invasive access for monitoring and intervention. ISF access is illustrated via passive uptake into hydrogel-forming MNs and higher-volume collection using an external vacuum patch applied over MN-created micropores. The sensing module supports multimodal transduction and time-resolved readouts of core state variables (pH, lactate, and oxygenation) with wireless display. Key drift drivers—motion, tissue response, and fouling film deposited on MN surfaces—are countered using reference/differential channels and algorithmic correction, enabling trajectory-based interpretation (rate of change, persistence, and multi-signal concordance). Therapeutic callouts depict MN penetration through a biofilm barrier and staged/triggered local delivery using internal (pH/redox/enzyme) or external (light/electric) cues to support practical decision-guided “sense–treat” wound management.

Microneedle sensors are increasingly described as “dermal access ports” that translate ISF into time-resolved readouts while maintaining a minimally invasive, wearable interface (Figure 2). For CLTI-related wounds, this format is attractive because it supports serial tracking of state transitions rather than single-point snapshots that may miss rapid microenvironmental drift (Li et al., 2024).

Microneedle access to ISF can be achieved through either passive diffusion-driven uptake or externally assisted extraction. Hydrogel-forming MNs swell *in situ* and can collect or shuttle dermal ISF without traditional pumping, which enables simpler wearable designs and may allow longer on-skin residence times (Aroche et al., 2025). A complementary approach creates micropores with MNs and then uses an external driving force. In this strategy, microneedle-created pores are paired with vacuum-assisted patches. This design can rapidly harvest higher-volume ISF for multiplex assays, such as proteomics or immunoassays (Jiang et al., 2024). Across platforms, sampling performance depends on insertion reliability, sampling kinetics, and local tissue reaction. Therefore, reporting standards for depth, dwell time, extraction volume, and skin-site variability are essential for interpretability (Saifullah and Faraji Rad, 2023).

Most microneedle-monitoring systems couple the microneedle interface to electrochemical, optical, or colorimetric transduction, but clinical utility depends on end-to-end integration, including calibration, electronics, wireless transmission, and user workflow (Tehrani et al., 2022). A recent example of “wearable-grade” integration demonstrated continuous ISF metabolite monitoring in freely behaving volunteers with smartphone-linked readout. This work emphasized the importance of device packaging and signal processing, not only needle chemistry (Tehrani et al., 2022). For chronic wound applications, integration of sensing with infection-control logic is emerging. Examples include microneedle patches that modulate local bioburden while visualizing wound status to support earlier escalation of care (Xiao et al., 2024).

### 3.1 Core sensing targets for CLTI-relevant state tracking

pH. pH is a practical state marker because it is directly measurable and influences enzyme activity and binding interactions that can distort other biosensor signals, making it both a biomarker and a calibration variable (Dervisevic et al., 2023). Microneedle-enabled pH monitoring has been implemented in wearable formats with potentiometric readout and strategies to reduce sweat interference. This is relevant for lower-limb wounds, where occlusion and exudate can confound surface measurements (Dervisevic et al., 2023). Low-cost pH monitoring concepts are also being explored using microneedle-enabled colorimetric readouts with smartphone analysis. These approaches may suit home monitoring if accuracy and robustness to lighting and skin tone are addressed (Kadian et al., 2024).

Lactate. Lactate is a high-value “metabolic stress” marker for ischemic tissue because it reflects glycolytic load and perfusion–oxygen mismatch, and it is technically feasible for continuous microneedle sensing using enzymatic electrochemistry (Ming et al., 2022). A first-in-human phase I study validated continuous lactate tracking via a minimally invasive microneedle patch with dynamic agreement to venous lactate trends, supporting feasibility for longitudinal metabolic monitoring outside intensive sampling workflows (Ming et al., 2022).

Oxygenation. Oxygenation is a central prognostic axis in ischemic tissue loss, yet surface-only approaches can miss deeper or rapidly changing states. This supports interest in minimally invasive sensing closer to the microvascular compartment (Jiang et al., 2026). A luminescent microneedle sensor array has been reported for *in situ* tissue oxygenation monitoring in diabetic foot ulcer models, illustrating how optical lifetime-based sensing can be packaged into a pain-minimized wearable form factor (Jiang et al., 2026).

### 3.2 Beyond the core panel

Beyond pH, lactate, and oxygenation, ISF targets may be extended to protein biomarkers and other molecular classes when sampling volume, stability, and contamination control permit. Microneedle patches have enabled ultrasensitive protein quantification in ISF (Wang et al., 2021), and microneedle concepts have been explored for nucleic acid capture and sensing (Al Sulaiman et al., 2019). Aptamer-based microneedle electrochemical sensors have also demonstrated continuous molecular monitoring in dermal ISF, suggesting potential for drug-level tracking and linking delivered therapy to measured state response (Friedel et al., 2023).

### 3.3 Calibration, drift, and interpretability

Signal drift is a non-negligible design constraint for microneedle sensors. It is often influenced by local tissue responses, electrode fouling, and motion artifacts. Accordingly, device studies increasingly emphasize differential sensing, reference channels, or algorithmic correction rather than raw amperometry alone (Yang et al., 2024). For CLTI-oriented use, the case for microneedle monitoring is strongest when the device functions as a state estimator: it supports reliable trajectory detection (rate of change, persistence, and multi-signal concordance) in a setting where ischemic and infectious signatures can overlap (Hossain et al., 2024). In practice, this framing shifts the performance bar from single-timepoint accuracy to long-wear stability, trend confidence, and workflow-resilient calibration under dressing changes and variable exudate.

## 4 Microneedle-enabled local therapy and triggered “sense–treat” design patterns

In ischemic wounds and ulcers, topical antimicrobials and biologics are often diffusion-limited by necrotic slough, exudate, and polymicrobial biofilm barriers (Haidari et al., 2024). Microneedle delivery can physically bypass this surface resistance and place payloads closer to viable peri-wound tissue, increasing the likelihood that local concentrations reach a therapeutic window. In the wound setting, microneedle arrays are increasingly positioned as “biofilm-penetrating, localized depots” that can raise effective drug exposure while reducing systemic burden. For CLTI, this framing aligns with the clinical reality that infection control and regeneration are coupled and often require staging rather than being treated as independent tasks. Three transferable “sense–treat” design patterns are discussed for ischemic, infection-prone tissue; evidence is drawn largely from wound-relevant models, and clinical relevance in CLTI depends on safety, stability, and alignment with limb-salvage workflows.

### 4.1 Anti-infective microneedle modules as state stabilizers

A practical pattern combines mechanical biofilm traversal with localized bactericidal chemistry (W et al., 2021). For example, flexible polymer-composite microneedle arrays can co-deliver oxygen and bactericidal agents to improve oxygenation while killing Gram-positive and Gram-negative bacteria in biofilm-like contexts. Another commonly used pattern is multimodal killing coupled with immune microenvironment shaping (Li et al., 2023). Infected-wound microneedle systems that combine photothermal and chemodynamic effects with pro-resolution immune modulation (for example, M2 polarization) are often presented as approaches that may shorten “infection to inflammation lock-in” and support subsequent granulation and re-epithelialization.

Some biofilms may also benefit from controlled “pulses” of killing to align with dressing-change cycles and reduce overtreatment. In this context, on-demand systems such as NIR-triggered antimicrobial peptide release from microneedle patches can be framed as examples of temporally programmable antibiofilm therapy (Su et al., 2023). As a CLTI-oriented design interpretation, these anti-infective modules are most defensible when described as state stabilizers: they aim to reduce bioburden and inflammatory amplification so that endogenous or delivered pro-repair cues can act in a more permissive niche.

### 4.2 Compartmentalization and staged release to match phase transitions

A second transferable pattern is compartmentalization, in which payloads are spatially arranged within the microneedle structure, most commonly as tip versus base loading, to encode timing and depth of delivery (Vora et al., 2023). In ischemic ulcers, tip-localized payloads can be optimized for rapid deposition into viable peri-wound tissue (e.g., an early antibiofilm or anti-inflammatory pulse), whereas base-resident cargo can support sustained exposure at the surface to reduce recolonization and maintain barrier protection (Long et al., 2023).

This staged logic aligns with infection–regeneration coupling in CLTI. Prolonged exposure to subtherapeutic antimicrobial levels is undesirable for biofilm control, and pro-regenerative signals such as growth factors may be more effective when delivered later, after the niche has been cleared and stabilized (Zhang et al., 2025). At the formulation level, many microneedle platforms explicitly target burst and maintenance (peak–hold pharmacokinetics) to avoid long tails of low drug levels. This principle is emphasized across long-acting microneedle delivery designs intended to improve efficacy while limiting resistance selection pressure (Vora et al., 2023). In practice, compartmentalization is also helpful for maintaining interpretability when sensing and delivery are co-located, because payload chemistry can otherwise perturb local pH or redox state.

### 4.3 Practical triggered actuation rather than full autonomy

Closed-loop microneedle systems can be framed as a three-step workflow: sensing the state variable, deciding or triggering an action, and delivering locally (Jian et al., 2025). For CLTI translation, the most defensible “closed-loop” framing emphasizes practical actuation rather than fully autonomous therapy (Teymour et al., 2021). Escalation can be triggered by thresholds or trends—for example, clinician alerts, intensified local anti-infective dosing, or dressing-change recommendations. This approach mirrors how closed-loop paradigms in other diseases are often introduced clinically, first as decision support with triggered dosing and only later moving toward autonomy, which may reduce regulatory and safety barriers in high-risk ischemic limbs (Yang et al., 2022).

Technically, triggers can be implemented through internal cues, such as pH-, redox-, or enzyme-responsive depots, or through external cues, such as light or electric fields (Qi et al., 2023). Trigger thresholds can be grounded in a compact panel that includes oxygenation, lactate, and pH with optional temperature (Qi et al., 2023). As proof-of-concept for “state variable to actuation,” wound-management demonstrations integrate microneedle electrodes for biosensing with microneedle-enabled drug delivery in the same platform, illustrating how parameters such as impedance can be translated into intervention rules (Fu et al., 2026). To minimize false triggers, CLTI-oriented designs should emphasize trajectory detection—rate of change, persistence, and multi-signal concordance—rather than single-point cutoffs (Ming et al., 2022).

### 4.4 Design principle: preserving sensing interpretability during co-therapy

A pragmatic “sense–treat” blueprint for CLTI uses a parallel architecture that physically separates a sensing microneedle sub-array from a delivery microneedle sub-array so that the readout reflects tissue state rather than drug chemistry (Zhao et al., 2025). This separation is particularly important when the therapeutic depot includes redox-active antimicrobials, catalytic nanozymes, or pH-modulating excipients, which can shift electrochemical baselines and create false trends if colocated with the sensor (Haidari et al., 2024). In chronic limb wounds, exudate and dressing changes can introduce intermittent perturbations. Under these conditions, a separated sensing zone can be protected by semi-permeable membranes or microfluidic routing to buffer mechanical shear and reduce contamination during clinical handling (Ahmadpour et al., 2023). More broadly, wearable-grade microneedle system integration has been strengthened by platforms that combine microneedle arrays, on-board electronics, and wireless pipelines for continuous ISF monitoring, providing a practical template for CLTI-oriented packaging (Tehrani et al., 2022).

## 5 Discussion

Building on the preceding sections, the central question for CLTI is not whether microneedle sensing or microneedle delivery can function in skin, but whether an integrated, triggered “sense–treat” system can remain interpretable, stable, and safe under chronic ischemia while producing outcomes that matter for limb salvage pathways (Conte et al., 2019). Dermal ISF microneedle platforms have progressed beyond glucose-only paradigms to multi-analyte, wearable-grade systems supported by advances in microneedle designs, transduction chemistries, and on-body electronics (Kim et al., 2024). Clinical feasibility for continuous metabolite tracking using integrated microneedle arrays has been demonstrated in humans, with wireless, real-time operation, indicating that the “sampling + sensing + transmission” stack is sufficiently mature to be repurposed for CLTI-oriented markers (Tehrani et al., 2022). On the delivery side, microneedle depots in dermatology and wound contexts provide credible local-delivery building blocks, and infection-facing designs increasingly incorporate antibiofilm intent and tissue-compatible biomaterials rather than relying on topical exposure alone (Haidari et al., 2024).

Key barriers for CLTI translation include drift and calibration during chronic wear, safety in fragile ischemic skin, and workflow fit. Oxygenation sensing and stability during chronic wear remain rate limiting because ischemic limbs impose low-perfusion chemistry, exudate, and high variability that challenge calibration and wear time. Longitudinal drift driven by biofouling and tissue reactions is repeatedly identified as a dominant barrier for microneedle sensors, which is particularly consequential when decisions are based on trends rather than single time points (Ben Halima et al., 2025). Accordingly, near-term CLTI systems should incorporate anti-fouling and stabilization strategies as primary design deliverables (Zhou et al., 2024). Examples include hydrogel interfaces, zwitterionic layers, recessed cavities, and redundant referencing, which have begun to show tangible performance gains in microneedle electrochemical sensing (Zhou et al., 2024).

From a clinical workflow perspective, the value of a microneedle-based monitoring panel may be greatest when it supports decisions between visits rather than replacing standard vascular assessment. For example, persistent deterioration in local oxygenation together with rising lactate and adverse pH drift may indicate an unfavorable wound trajectory before overt tissue breakdown becomes evident. In addition, concordant worsening across these signals may help prompt earlier reassessment for infection escalation or wound instability. After revascularization, debridement, or infection-control treatment, repeated trend-based measurements may also provide a practical way to evaluate whether the wound microenvironment is shifting toward a more permissive healing state.

Finally, CLTI management is episodic and multidisciplinary, so devices must tolerate dressing changes, exudate, and variability in outpatient behavior. In this context, the practical value of microneedle monitoring is strongest when it supports timely escalation between visits, and the practical value of microneedle therapy is strongest when it is staged and aligned with clinical routines (e.g., dressing intervals and infection workups). Assay complexity that disrupts workflow will compete directly with adoption, even if analytical performance is strong (Poudineh, 2024).

In summary, microneedle-enabled monitoring and localized delivery can be framed as complementary adjuncts for CLTI. The strongest near-term rationale centers on trend-based state tracking and staged, infection-aware intervention rather than exhaustive biomarker coverage or fully autonomous actuation.Across the concepts reviewed here, translation depends on maintaining interpretability under chronic ischemia, including drift mitigation and robust calibration. It also depends on meeting non-negotiable safety constraints in fragile limb skin and fitting real-world wound-care workflows.These considerations suggest that minimally sufficient sensing of pH, lactate, and oxygenation, paired with phase-matched local modules, may offer a practical path toward clinically actionable “sense–treat” systems for threatened limbs.

## 6 Conclusion

Microneedle platforms may serve as state-aware interfaces for CLTI by supporting trend-based monitoring together with staged local therapy. A practical translational strategy is to prioritize a minimal actionable sensing panel centered on pH, lactate, and tissue oxygenation, with temperature added when clinically needed. Rather than pursuing exhaustive biomarker coverage, near-term systems may be more effective when coupled to phase-matched local interventions and designed for long-wear stability, safety in fragile ischemic skin, and compatibility with real-world wound-care workflows.
